# Genetic Diversity and Population Structure of Indian Golden Silkmoth (*Antheraea assama*)

**DOI:** 10.1371/journal.pone.0043716

**Published:** 2012-08-28

**Authors:** Kallare P. Arunkumar, Anup Kumar Sahu, Atish Ranjan Mohanty, Arvind K. Awasthi, Appukuttannair R. Pradeep, S. Raje Urs, Javaregowda Nagaraju

**Affiliations:** 1 Centre of Excellence for Genetics and Genomics of Silkmoths, Laboratory of Molecular Genetics, Centre for DNA Fingerprinting and Diagnostics, Hyderabad, India; 2 Regional Muga Research Station, Central Muga Eri Research and Training Institute, Boko, India; 3 Seribiotech Research Laboratory, Central Silk Board, Bangalore, India; University of Oxford, United Kingdom

## Abstract

**Background:**

The Indian golden saturniid silkmoth (*Antheraea assama*), popularly known as muga silkmoth, is a semi-domesticated silk producing insect confined to a narrow habitat range of the northeastern region of India. Owing to the prevailing socio-political problems, the muga silkworm habitats in the northeastern region have not been accessible hampering the phylogeography studies of this rare silkmoth. Recently, we have been successful in our attempt to collect muga cocoon samples, although to a limited extent, from their natural habitats. Out of 87 microsatellite markers developed previously for *A. assama*, 13 informative markers were employed to genotype 97 individuals from six populations and analyzed their population structure and genetic variation.

**Methodology/Principal Findings:**

We observed highly significant genetic diversity in one of the populations (WWS-1, a population derived from West Garo Hills region of Meghalaya state). Further analysis with and without WWS-1 population revealed that dramatic genetic differentiation (global F_ST_ = 0.301) was due to high genetic diversity contributed by WWS-1 population. Analysis of the remaining five populations (excluding WWS-1) showed a marked reduction in the number of alleles at all the employed loci. Structure analysis showed the presence of only two clusters: one formed by WWS-1 population and the other included the remaining five populations, inferring that there is no significant genetic diversity within and between these five populations, and suggesting that these five populations are probably derived from a single population. Patterns of recent population bottlenecks were not evident in any of the six populations studied.

**Conclusions/Significance:**

*A. assama* inhabiting the WWS-1 region revealed very high genetic diversity, and was genetically divergent from the five populations studied. The efforts should be continued to identify and study such populations from this region as well as other muga silkworm habitats. The information generated will be very useful in conservation of dwindling muga culture in Northeast India.

## Introduction

Comparison of genetic structure among populations of a species, which have carved their own niche in a narrow ecological range, can illuminate our understanding of environmental factors affecting their adaptation and selection forces operating on them to retain such species for a long time [1], [2]. The Northeast India is recognized as one of the 34 identified ‘hot spots’ of biodiversity in the world, which is endowed with a vast variety of flora and fauna [3]. The Indian golden silkmoth (*Antheraea assama*, Lepidoptera: Saturniidae), popularly known as muga silkmoth, is a semi-domesticated, golden colored silk producing insect, endemic to Northeast India [4]. It is a polyvoltine and polyphagous insect that feeds on 15 different host plant species. The shimmering golden yellow silk has been mentioned in the great Hindu epic ‘Mahabharata’ as a constituent of ‘Peetambaram’, the yellow silk garment. Historical records also suggest the presence of muga silk in Northeast India since 321 BC. Mentions about this insect can be found in the literature as early as 1662 BC [4]. This luminous golden muga silk has now secured Geographical Indications (GI) status, the recognition under the intellectual property rights that it has its origin in the Assam region of the Northeast India. Because of its commercial exploitation and deforestation activities resulting in depletion of its host plant populations, the population density of this silkmoth is reported to be showing a declining trend [5]. Empirical observations suggest that genetic variation of this species is low because of the rapid decline in population density [5]. But there is no direct evidence to test this and phylogeography of this species has hardly been investigated either at ecological or molecular level. Only recently a set of microsatellite markers was isolated for this species [6].

Ecological research on insect species provide invaluable information on population structure, speciation, gene flow and genetic diversity, and offers an explanation on insect diversity based on their interaction with environmental factors, either biotic (including other species) or abiotic. Molecular marker data help to distinguish populations of a species as well as taxonomic relationships of a species in question [7]. In insects, DNA markers are used to provide information based on which estimates of genetic diversity and gene flow between populations can be obtained, and migration and colonization history can be analyzed [8], [9], [10], [11]. Among DNA markers, microsatellite or simple sequence repeat (SSR) markers have proven potential in diversity analysis owing to their co-dominant nature, high level of polymorphism, amenability to high throughput analysis and as informative markers to address population genetics questions in a given species [12].

Recently, we reported 12 informative SSR markers, in a muga silkworm population, out of 87 derived from Expressed Sequence Tags (EST-SSRs) and a repeat enriched genomic library (genomic-SSRs) [6]. In the study presented here we have used 11 of these and 2 newly characterized genomic–SSRs to assess the genetic variability in 6 populations of *A. assama* collected from different locations of Northeast India. We have also attempted to analyze the genetic structure and test for signs of a bottleneck in these populations.

## Results and Discussion

**Figure 1:**
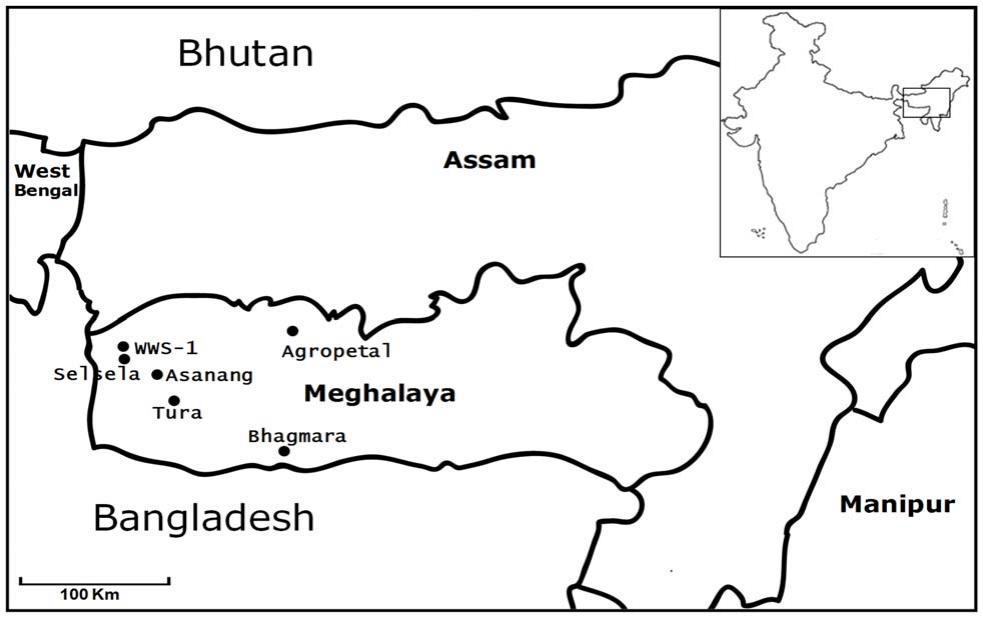
Sampling locations of muga silkmoth, *A. assama*.

In the present study, we genotyped 97 individuals from 6 populations (Figure 1) of *A. assama* with 13 informative microsatellite markers [6] (Table S1) to understand the population structure and to estimate the genetic variability.

### Polymorphism and Allele Frequency

Among 13 polymorphic microsatellite markers used on 6 populations, five loci showed within population polymorphism in all the populations (Table S1). Number of alleles among polymorphic loci ranged from 2 to 8. We found 59 alleles for thirteen microsatellite loci in this study, across all the six populations. The most diverse locus (AaSat020) had 8 alleles and the least diverse ones (AaSat006, AaGSat019 and AaGSat037) had 2. Information on the number of alleles (Na), number of effective alleles (Ne), observed (Mean Ho) and expected (Mean He) mean heterozygosities, fixation index (F) and F statistics polymorphism by population is presented in Table 1. The presence of null alleles at each locus was tested using MICROCHECKER. Three loci AaSat002, AaSat044 and AaSat065 exhibited overall significant excess of homozygotes, possibly indicating the presence of null alleles.

**Table 1:** Locus-wise data on the number of alleles (Na), number of effective alleles (Ne), mean observed (Mean Ho) and expected (Mean He) heterozygosities, the number of migrants (Nm), fixation index (F) and F statistics.

Locus	Na	Ne	Mean Ho	Mean He	Nm	F		F_IS_	F_IT_	F_ST_
**AaSat001**	4	1.170	0.021	0.097	0.164	0.868		0.784	0.915	0.604
**AaSat002**	7	2.350	0.173	0.493	0.933	0.713		0.649	0.723	0.211
**AaSat006**	2	1.195	0.068	0.111	0.484	0.110		0.386	0.595	0.340
**AaSat008**	4	1.059	0.039	0.044	2.533	0.105		0.105	0.186	0.090
**AaSat14**	3	1.232	0.173	0.158	0.187	0.094		−0.094	0.532	0.572
**AaSat020**	8	1.410	0.089	0.156	0.262	0.436		0.432	0.709	0.488
**AaSat040**	7	2.249	0.202	0.454	0.858	0.546		0.555	0.655	0.226
**AaSat044**	5	1.537	0.132	0.335	0.660	0.540		0.606	0.714	0.275
**AaSat053**	6	1.616	0.161	0.298	0.639	0.479		0.458	0.610	0.281
**AaSat065**	5	1.347	0.039	0.233	0.417	0.897		0.832	0.895	0.375
**AaGSat019**	2	1.074	0.000	0.063	5.558	1.000		1.000	1.000	0.043
**AaGSat026**	4	1.126	0.013	0.079	1.485	0.480		0.839	0.862	0.144
**AaGSat037**	2	1.295	0.181	0.177	0.697	−0.058		−0.024	0.246	0.264
**Mean**							0.499	0.502	0.665	0.301

Information on percentage polymorphism, number of private alleles, number of alleles per locus etc., of six populations under study is presented in Table 2. Private alleles were present in three populations (Tura, Asanang and WWS-1), although the number varied between populations, with WWS-1 showing the highest number of private alleles (Table 2 and Table S2). Linkage disequilibrium studies after sequential Bonferroni correction revealed that none of the loci were significantly associated with each other, thereby allowing the use of standard algorithms of population genetics for data analysis.

**Table 2:** Population-wise data on the mean number of alleles per locus (Na), number of effective alleles (Ne), number of private alleles, mean observed (Ho) and expected (He) heterozygosity, fixation index (F) and percentage polymorphism observed in 13 SSR loci (% P).

Population	Na	Ne	Ho	He	No. private alleles	F	% P
WWS-1	4.000	2.362	0.252	0.474	1.923	0.553	100
Selsela	1.692	1.303	0.107	0.173	0.000	0.365	46.15
Asanang	1.923	1.248	0.099	0.162	0.077	0.275	61.54
Tura	1.923	1.232	0.050	0.139	0.231	0.436	61.54
Agropetal	1.923	1.277	0.075	0.178	0.000	0.536	76.92
Bhagmara	1.462	1.190	0.012	0.120	0.000	0.901	38.46

F_IS_ (inbreeding coefficient) is the proportion of variance in the subpopulation contained in an individual. In the present analysis we observed high F_IS_ values when all the six populations were considered (F_IS_ = 0.502) (Table 1) and also when only five populations were considered for analysis (F_IS_ = 0.447) (Table 3). High F_IS_ indicated a high degree of inbreeding among individuals within populations. This is in contrast to previous reports on population genetic studies in lepidopterans, which show the F_IS_ to be low in most of the species studied, e.g., *Cydia pomonella* [13], *Parnassius mnemosyne* [14], *Ostrinia nubilalis* [15] and *Erebia euryale* [16].

**Table 3:** Locus-wise data on the number of alleles (Na), number of effective alleles (Ne), mean observed (Mean Ho) and expected (Mean He) heterozygosities, the number of migrants (Nm), fixation index (F) and F statistics obtained by analyzing five populations excluding WWS-1 population.

Locus	Na	Ne	Mean He	Mean Ho	Nm	F	F_IS_	F_IT_	F_ST_
**AaSat001**	2	1.023	0.021	0.000	5.313	1.000	1.000	1.000	0.045
**AaSat002**	4	1.831	0.432	0.090	1.191	0.803	0.791	0.828	0.174
**AaSat006**	2	1.036	0.034	0.035	19.579	−0.030	−0.031	−0.018	0.013
**AaSat14**	3	1.250	0.165	0.208	1.831	−0.208	−0.260	−0.108	0.120
**AaSat020**	4	1.069	0.052	0.029	2.869	0.446	0.446	0.490	0.080
**AaSat040**	2	1.702	0.385	0.101	0.835	0.632	0.736	0.797	0.230
**AaSat044**	3	1.514	0.323	0.135	3.122	0.508	0.583	0.614	0.074
**AaSat053**	4	1.341	0.224	0.147	3.523	0.437	0.346	0.389	0.066
**AaSat065**	3	1.249	0.189	0.000	7.203	1.000	1.000	1.000	0.034
**AaGSat019**	2	1.065	0.055	0.000	4.297	1.000	1.000	1.000	0.055
**AaGSat026**	2	1.016	0.015	0.015	7.813	−0.040	−0.040	−0.008	0.031
**AaGSat037**	2	1.154	0.112	0.135	2.047	−0.136	−0.203	−0.072	0.109
				**Mean**	4.586	0.480	0.447	0.493	0.086

The degree of correspondence between the geographic distance matrix and genetic distance matrix was analyzed using Mantel test [17]. The correlation coefficient was calculated to be −0.34 with an associated probability of 0.12. This shows that there is no real evidence of a relationship between genetic and spatial distance of different populations.

### Departure from Hardy–Weinberg Equilibrium (HWE)

The observed and expected heterozygosities varied depending on the microsatellite loci. Eighteen of the 50 population-loci combinations showed no significant departures from HWE (Table S3). Three of the eight loci polymorphic in Tura, three of thirteen microsatellite markers in WWS-1, four of seven polymorphic markers in Asanang and three of ten polymorphic loci in Agropetal population showed no significant deviation from HWE. In Selsela population only 2 out of 6 polymorphic loci deviated from HWE. Only in Bhagmara population all the 5 polymorphic loci deviated from HWE. Taking the possible presence of null alleles in three loci (AaSat002, AaSat044 and AaSat065) into consideration, the results suggest that the deviation from HWE of many loci may be due to presence of null alleles and smaller sample size used in the analysis.

### Population Structure

**Figure 2:**
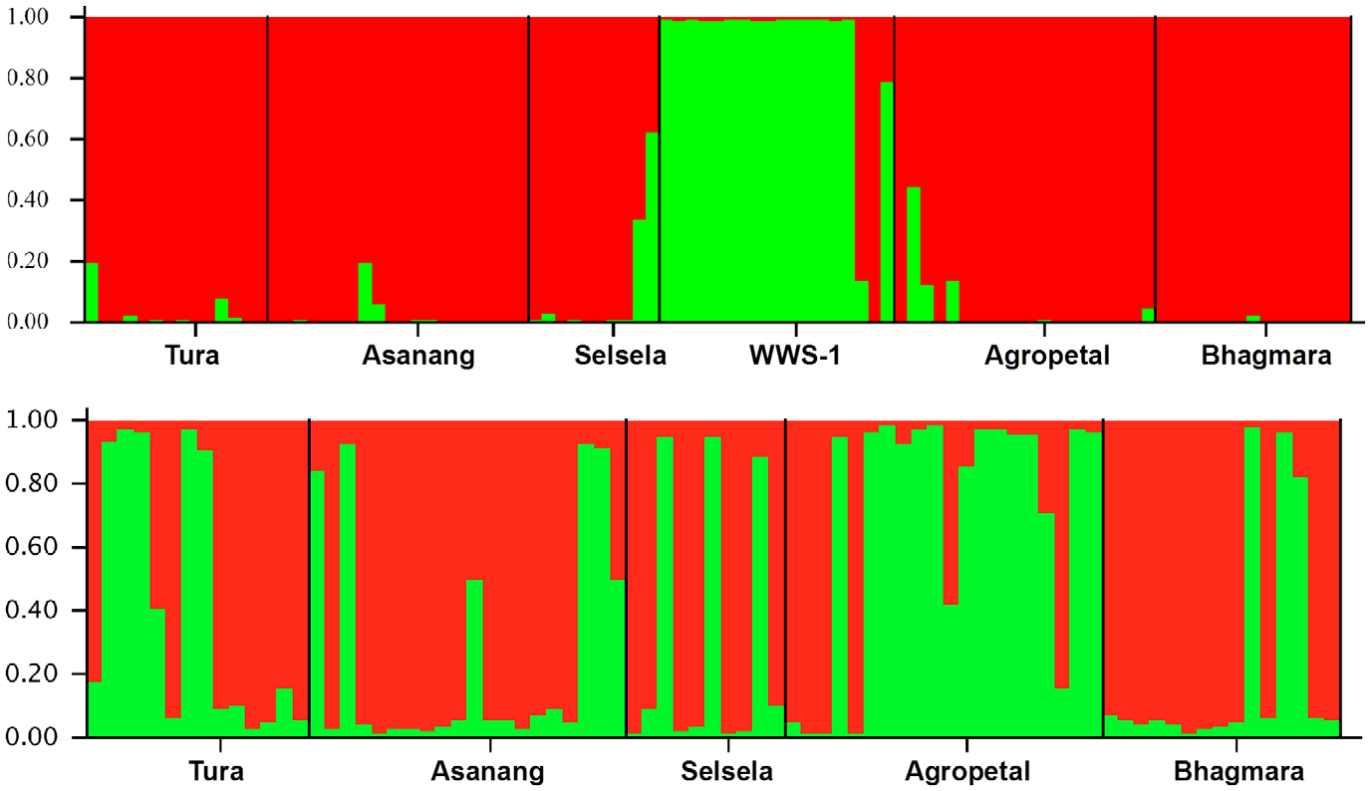
Population structure of six *A. assama* populations prepared using STUCTURE program. Upper panel shows the structure obtained by analysis of all six populations and the lower panel shows the structure obtained by excluding WWS-1 population.

Population structure was investigated using the STRUCTURE program to estimate the number of genetically distinct populations (K). To calculate the most appropriate K value formed by the 97 individuals, we utilized an *ad hoc* statistical analysis based on the second-order rate of change of the likelihood function with respect to K (ΔK), as described previously [18] using the software program Structure Harvester v0.6.92 [19]. There was a clear peak in the value of ΔK, as determined by the method of Evanno et al. [18], at K = 2. This indicated the presence of 2 clusters clearly differentiating the WWS-1 from the rest of the populations (Figure 2).

All the populations except WWS-1 formed one cluster and WWS-1 formed a separate cluster. As all the six populations are less than 100 km apart from each other and as the *A. assama* moths can fly for up to 20 Km distance, there seems to be migration between populations. This is evident from the structure analysis that showed shared genetic clusters between populations. Though there was no big physical barrier that restricts the migration of *A. assama* from WWS-1 region, we speculate that the presence of bamboo thicket and tall trees (such as *Shorea* and *Tectona* species) around this region may act as a barrier, thus limiting the migration of *A. assama* from the WWS-1 region.

Since WWS-1 was found to form a separate cluster in STRUCTURE analysis, we carried a of population structure analysis excluding WWS-1. The five populations did not show any distinct structure to suggest that they are individual populations. The analysis revealed the presence of two sub-clusters in five populations, indicating that there is a mix of two populations (Figure 2).

**Figure 3:**
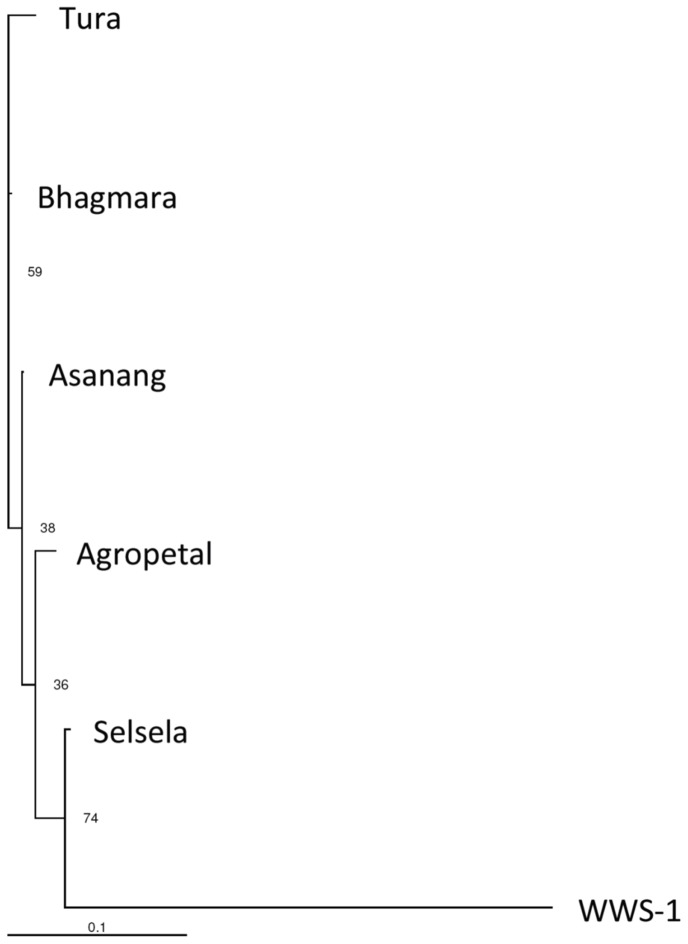
NJ tree constructed based on Nei minimum genetic distance matrix data. The numbers near the nodes are the bootstrap values.

A neighbour joining (NJ) tree was constructed using Populations program with Nei’s minimum genetic distance matrix values (Figure 3). Populations sampled from adjacent locations showed low genetic divergence, compared to locations far apart. WWS-1 showed extended branch length. Structure analysis and NJ tree indicate that WWS-1 is genetically the most diverse population and is genetically divergent from the other five populations. Analysis of data considering individuals of each of the populations revealed the clustering of most of the WWS-1 individuals into one group and most of the individuals from the remaining five populations forming another cluster (Figure S1).

AMOVA was calculated for all the six populations and also separately for five population, excluding WWS-1, to examine the effect of highly diverse population WWS-1 on overall variability. AMOVA revealed that variation among populations (34%, variance component 0.77) was almost same as that observed among individuals within populations (36%, variance component 0.81) when all the six populations were considered for analysis. Interestingly, when only five populations were considered (excluding WWS-1), among populations variation was found to be only 9% (variance component 0.11), which is far less than that observed among individuals within populations (53%, variance component 0.63). Variation within individuals was similar when WWS-1 was included (30%, variance component 0.67) and when it was excluded (37%) from the populations. These results indicate that high among population diversity was due to genetically diverse population, WWS-1.

### Bottleneck Analysis

Populations that have experienced a recent reduction in their effective population size exhibit a correlated reduction in allele numbers and heterozygosities at polymorphic loci. It is expected that the allelic diversity is reduced faster than the heterozygosity, i.e. the observed heterozygosity is larger than the heterozygosity expected from the observed allele number where the locus is at mutation-drift equilibrium [20]. We analyzed the allele frequencies using the program BOTTLENECK [20]. With fewer than 20 loci, the Wilcoxon test is shown to be robust [21] so this was chosen as the test of choice. The analysis revealed no apparent genetic bottleneck in any of the 6 populations. Except Tura population that showed a significant heterozygote deficiency (*P* = 0.009), all the other populations did not show any significant heterozygote deficiency or excess. This heterozygote deficiency may be due to presence of null alleles, as our analysis revealed possible occurrence of null alleles in three of the loci tested.

Low between population genetic diversity and high within population diversity, and lack of apparent population structure and higher number of shared alleles between 5 populations (excluding WWS-1) indicate that the five populations excluding WWS-1 may indeed constitute a single population.

### Conclusion

This is the first known report describing population genetics of *A. assama*. Our analysis showed no real evidence of a significant correlation between genetic and spatial distance of different populations. Among the six populations examined in this study, WWS-1 population showed very high genetic diversity and was genetically divergent from the remaining five populations. On the other hand, the five populations, which we had collected from different regions were quite similar and are likely to have been founded from a single population. The *A. assama* populations like WWS-1, can be utilized in the conservation efforts of the muga silkworm. Though present analysis gave a reasonable insight into population genetics of *A. assama*, the analysis with a bigger sample size and including many more forest-based population, would give a much larger picture. However, difficulty in collecting more samples owing to hostile conditions particularly inaccessible forest terrains, politically sensitive border areas and prevailing insurgency conditions is hampering further studies on population genetics of this silkmoth.

Since the sampling is only from a part of the Northeast Indian region inhabited by this silkmoth, it may not account for the genetic variability existing in the populations in other regions. Efforts are being made to collect insect samples from other regions. These efforts, if successful, would give a clear picture of population structure and other details of population variability of *A. assama* in whole of Northeast India.

## Materials and Methods

### Sampling, DNA Extraction and PCR

A total number of 97 nature grown pupae of *A. assama* were collected from six different geographical locations of Northeast India (Figure 1 and Table 4). In each area the cocoons were collected from host plants, within the forest area of about 500 m radius. No specific permits were required for the collection, as *A. assama* is not an endangered or protected species. However, sampling was very difficult as the forest area was not easily accessible due to inhospitable terrains and prevailing insurgent activities in that region. Therefore screening was done with the limited samples collected. Individuals collected from a particular location were considered as a single population in the following analysis and given common geographic co-ordinates (Table 4). Genomic DNA was isolated from each pupa separately, by grinding in liquid nitrogen, using the method described earlier [22].

**Table 4:** *A. assama* populations screened with SSR markers, their place of collection and number of individuals screened in each population.

Sl. no.	*A. assama* population	Place of collection	Sampling coordinates	Number of individuals
1	WWS-1	West Garo Hills, Meghalaya	25.82N/90.00E	18
2	Selsela	Bolsalgere, Selsela, West Garo Hills, Meghalaya	25.77N/90.00E	10
3	Asanang	Digrongre, Asanang, West Garo Hills, Meghalaya	25.68N/90.15E	20
4	Tura	Tura, Meghalaya	25.51N/90.26E	14
5	Agropetal	East Garo Hills, Meghalaya	25.86N/90.88E	20
6	Baghmara	Tinang, Bagmara, South Garo Hills, Megahalya	25.22N/90.79E	15

The primers were labelled at 5′ end using two different fluorescent dyes, namely TAM (yellow) and FAM (blue) (Table S1). All polymerase chain reactions (PCR) were carried out in a 10 ul reaction mixture containing 1X PCR buffer (Applied Biosystems, USA), 0.1 mM dNTPs, 1.0 to 3.0 mM MgCl_2_, 5 picomole of each primer, 10 ng of genomic DNA as template and 0.5 U AmpliTaq Gold™ DNA Polymerase (Applied Biosystems). The reactions were carried out in a Vapo-protect Gradient thermal cycler (Eppendorf, Germany). The PCR conditions were; 94°C for 3 minutes as initial denaturation, and 35 cycles of: 94°C denaturing for 20 seconds, the appropriate annealing temperature for 10 seconds and 72°C extension for 45 seconds and 72°C for 10 minutes as final extension. All the amplicons were run on an ABI PRISM 3730 DNA analyzer, with Pop-7 as sieving matrix, HiDi Formamide as single-stranded DNA stabilizer and GeneScan 500 ROX standard as a size marker. ABI PRISM GeneMapper software version 3.0 was used to size the alleles.

### Data Analysis

The level of polymorphism was tested with 13 microsatellite markers, which were evaluated earlier for their informativeness, by genotyping all the individuals from six populations. For the EST-SSRs and genomic SSRs, mean number of alleles (*A*), percentage of polymorphic loci (*P*), mean unbiased estimates of genetic diversity (H*_E_*) [23] and observed heterozygosity (H*_O_*) were estimated using GenAlex 6 software [24]. Population allele frequencies were calculated using the program GenAlex 6 [24]. To test for significant isolation by distance, Mantel’s test [17] was performed with 10,000 permutations using the software zt Version 1.1 [25]. For this, the genetic distance matrix was calculated using GDA 1.1 [26], and geographical distance matrix was calculated using populations’ location co-ordinates (latitude and longitude) with Geographic Distance Matrix Generator [27]. The number of migrants per generation (Nm), a measure of historical gene flow, was estimated by the private allele method using GENEPOP 4.0 [28]. To determine the genetic relationships among these populations, a NJ dendrogram based on Nei’s minimum genetic distance was constructed, by carrying out 1000 bootstrap replications, using Populations program (http://bioinformatics.org/~tryphon/populations/). A NJ tree was also constructed on per individual basis to test whether individuals from WWS-1 form different cluster, using Populations software and considering Nei minimum distance method (http://bioinformatics.org/~tryphon/populations/).

Pairwise tests for linkage disequilibrium were performed using web-based program, GENEPOP version 3.4 [28]. The P values obtained were corrected using sequential Bonferroni correction. The probability of null allele occurrence was estimated using MICROCHECKER [29] in which null alleles were considered to occur at a locus if an overall significant excess of homozygotes is seen, distributed evenly across the homozygote classes. The microsatellite data were subjected to a hierarchical analysis of molecular variance (AMOVA), as described by Excoffier et al. [30], using three hierarchical levels; individual, population and inter-population level. The analysis was performed using Arlequin 2.000 [31].

Since WWS-1 showed very high genetic divergence within population whole data was reanalyzed, by removing WWS-1 population, to test if the results differ significantly.

### Bottleneck Analysis

The BOTTLENECK program [20] was used to determine if any signal of past bottleneck could be detected. Bottlenecked populations are predicted to show an excess of heterozygosity compared to the expected heterozygosity from allele frequencies, as the number of alleles is more severely affected than heterozygosity when a population undergoes bottleneck. BOTTLENECK was run under the two-phase model of microsatellite evolution [32] with 10% of the infinite allele model and 90% of the stepwise mutation model.

### Structure Analysis

Population structure was examined using the Bayesian clustering algorithm STRUCTURE 2.3.1 [33], [34], to estimate the number of potential genetic clusters (K) an individual could be placed in, based on their genotype. The program STRUCTURE facilitates testing the veracity of the hypotheses about the number of populations by calculating the probability of the data for each hypothesis. The program assumes that the markers are not in linkage disequilibrium and that they exhibit co-dominance. We ran STRUCTURE for 100,000 steps after a burn-in period of 100,000 steps with 20 replicate runs for each value of K (1 to 15). The population structure was analyzed assuming admixture in the population in correlated allele frequency model. Taking results from STRUCTURE output file, the number of true clusters in the data (K) was determined using Structure Harvester [19], which identifies the optimal K based on the posterior probability of the data for a given K, and the ΔK [18].

## Supporting Information

Figure S1 **Neighbour-joining tree of all the individuals, based on Nei minimum genetic distance matrix data.**
(DOCX)

Table S1 **Details of microsatellite loci used in this study (Arunkumar et al., 2009, Mol. Ecol. Res.).** Fluorescent labelling was done for only forward primers of all the loci.
(DOCX)

Table S2 **Summary of private alleles by population.**
(DOCX)

Table S3 **Summary of Chi-square tests for Hardy-Weinberg equilibrium.**
(DOCX)
