# Supplementary material for: Genetic Diversity and Population Structure of Indian Golden Silkmoth (Antheraea assama)
Source: PLoS One. 2012 Aug 28;7(8):e43716. doi: 10.1371/journal.pone.0043716 (PMC3429497; doi:10.1371/journal.pone.0043716)
Supplement: Figure S1 — Neighbour-joining tree of all the individuals, based on Nei minimum genetic distance matrix data. (DOCX) [file pone.0043716.s001.docx]

**
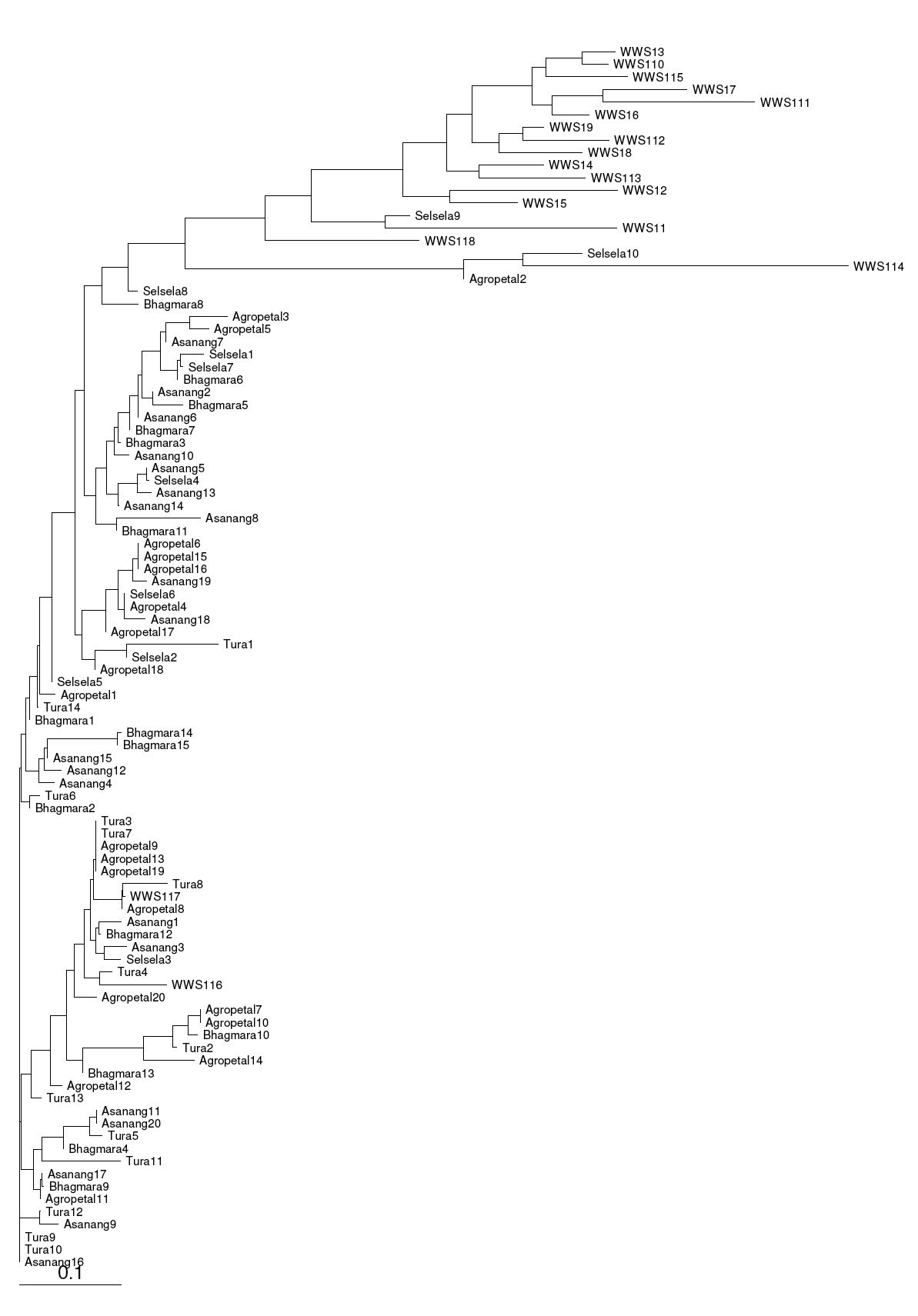
Figure S1: Neighbour-joining tree of all the individuals, based on Nei minimum genetic distance matrix data.**
