# Supplementary material for: Genetic Diversity and Population Structure of Indian Golden Silkmoth (Antheraea assama)
Source: PLoS One. 2012 Aug 28;7(8):e43716. doi: 10.1371/journal.pone.0043716 (PMC3429497; doi:10.1371/journal.pone.0043716)
Supplement: Table S1 — Details of microsatellite loci used in this study (Arunkumar et al., 2009, Mol. Ecol. Res.). Fluorescent labelling was done for only forward primers of all the loci. (DOCX) [file pone.0043716.s002.docx]

**Table S1: Details of microsatellite loci used in this study (Arunkumar et al., 2009, Mol. Ecol. Res.). Fluorescent labelling was done for only forward primers of all the loci.**

| **Locus** | **Fluorscent label** | **Source** | **Forward / Reverse primer (5’-3’)** | **Repeat motif** | **Size range** | **Annealing temp. (^O^C)** |
| --- | --- | --- | --- | --- | --- | --- |
| **AaSat001** | **FAM** | **EST** | GTGTTCATTTCACGGAACATT  CATTCGCTGTTCGTCTGAGAT | (TA)7 | 175-180 | 53 |
| **AaSat002** | **TAM** | **EST** | TCTGGACAAATTGTAAAAGCTGTAG  ACAAAACGAAAATCGCGTGT | (GTCT)5 | 120-139 | 51 |
| **AaSat006** | **TAM** | **EST** | ACGAGGCTGCAGTGAGATTT  AGGCTGATACGCAAGCTGAT | (TACG)5 | 213-215 | 56 |
| **AaSat008** | **FAM** | **EST** | CACGAAATGCCTCTGTCGTA  GGTGTCTGTGGATGATGTGC | (TA)6 Nn (CA)9 Nn (CA)5 | 188-211 | 51 |
| **AaSat14** | **TAM** | **EST** | ATCTCTACCTACGCCGACGA  AATTCGGCACGAGGAGTTC | (GAT)5 (TAA)4 | 464-470 | 48 |
| **AaSat020** | **FAM** | **EST** | TTTCTTCGGTTCGTTTGGTT  GACACGCGTTGCTTTGAGTA | (TCGTG)5 | 172-202 | 53 |
| **AaSat040** | **FAM** | **EST** | CGGACGTAACATTTGTCTGG  CCACATGACTCTCATCAGCA | (AT)17 | 106-130 | 60 |
| **AaSat044** | **FAM** | **EST** | CACCAGCTTCCAAAGAATTG CTAAAGCCCACGGGTTCATA | (AT)20 | 192-223 | 51 |
| **AaSat053** | **TAM** | **EST** | GAGTTCGGGTCGGACGTAAT  TCTCTACCTACGCCGACGAC | (ATT)4 N7 (ATC)6 | 202-225 | 50 |
| **AaSat065** | **TAM** | **EST** | GTCGAGCTGTCATAATTCCT  AGTCTGACGTCGCTATAACC | (AT)11 Nn (AT)11 (TA)5 | 98-118 | 54 |
| **AaGSat019** | **FAM** | **Genomic** | GATGGACTGGACCTCAATCG  CCTGAGGAGAGAGGCGATG | (TGA)2 (AGA)3 | 152-154 | 55 |
| **AaGSat026** | **FAM** | **Genomic** | GAAGTTGCGACTTGACACCA  TGCTTGATTTTTAATACTCGCTAGG | (GTAT)3 (GT)30 | 206-210 | 55 |
| **AaGSat037** | **TAM** | **Genomic** | CCCAACACATCTCCTCTGGT  AACATGGAGCGACAATCACA | (ACACT)3 .. (CA)5 | 132-243 | 55 |
