# Supplementary material for: Genetic Diversity and Population Structure of Indian Golden Silkmoth (Antheraea assama)
Source: PLoS One. 2012 Aug 28;7(8):e43716. doi: 10.1371/journal.pone.0043716 (PMC3429497; doi:10.1371/journal.pone.0043716)
Supplement: Table S2 — Summary of private alleles by population. (DOCX) [file pone.0043716.s003.docx]

**Table S2: Summary of private alleles by population**

| **Pop** | **Locus** | **Allele** | **Freq** |
| --- | --- | --- | --- |
| Tura | AaSat020 | 212 | 0.036 |
| Tura | AaSat044 | 193 | 0.071 |
| Tura | AaGSat026 | 216 | 0.038 |
| Asanang | AaSat014 | 462 | 0.031 |
| WWS1 | AaSat001 | 169 | 0.031 |
| WWS1 | AaSat001 | 177 | 0.063 |
| WWS1 | AaSat002 | 120 | 0.147 |
| WWS1 | AaSat002 | 122 | 0.029 |
| WWS1 | AaSat002 | 133 | 0.059 |
| WWS1 | AaSat008 | 186 | 0.029 |
| WWS1 | AaSat008 | 188 | 0.029 |
| WWS1 | AaSat008 | 197 | 0.088 |
| WWS1 | AaSat020 | 180 | 0.083 |
| WWS1 | AaSat020 | 187 | 0.056 |
| WWS1 | AaSat020 | 193 | 0.028 |
| WWS1 | AaSat020 | 202 | 0.528 |
| WWS1 | AaSat040 | 106 | 0.029 |
| WWS1 | AaSat040 | 108 | 0.147 |
| WWS1 | AaSat040 | 112 | 0.206 |
| WWS1 | AaSat040 | 116 | 0.059 |
| WWS1 | AaSat040 | 118 | 0.118 |
| WWS1 | AaSat044 | 202 | 0.059 |
| WWS1 | AaSat044 | 228 | 0.059 |
| WWS1 | AaSat053 | 221 | 0.441 |
| WWS1 | AaSat053 | 228 | 0.059 |
| WWS1 | AaSat065 | 109 | 0.029 |
| WWS1 | AaSat065 | 114 | 0.059 |
| WWS1 | AaGSat026 | 206 | 0.167 |
| WWS1 | AaGSat026 | 208 | 0.083 |
