# Supplementary material for: Genetic Diversity and Population Structure of Indian Golden Silkmoth (Antheraea assama)
Source: PLoS One. 2012 Aug 28;7(8):e43716. doi: 10.1371/journal.pone.0043716 (PMC3429497; doi:10.1371/journal.pone.0043716)
Supplement: Table S3 — Summary of Chi-square tests for Hardy-Weinberg equilibrium. (DOCX) [file pone.0043716.s004.docx]

**Table S3: Summary of Chi-square tests for Hardy-Weinberg equilibrium**

| **Pop** | **Locus** | **DF** | **ChiSq** | **Prob** | **Signif** |
| --- | --- | --- | --- | --- | --- |
| Tura | AaSat001 | Monomorphic | |  |  |
| Tura | AaSat002 | 3 | 14.002 | 0.003 | ** |
| Tura | AaSat006 | 1 | 0.019 | 0.890 | ns |
| Tura | AaSat008 | Monomorphic | |  |  |
| Tura | AaSat14 | Monomorphic | |  |  |
| Tura | AaSat020 | 6 | 14.097 | 0.029 | * |
| Tura | AaSat040 | 1 | 5.498 | 0.019 | * |
| Tura | AaSat044 | 3 | 19.437 | 0.000 | *** |
| Tura | AaSat053 | Monomorphic | |  |  |
| Tura | AaSat065 | 1 | 14.000 | 0.000 | *** |
| Tura | AaGSat019 | Monomorphic | |  |  |
| Tura | AaGSat026 | 1 | 0.021 | 0.885 | ns |
| Tura | AaGSat037 | 1 | 0.019 | 0.890 | ns |
| Asanang | AaSat001 | Monomorphic | |  |  |
| Asanang | AaSat002 | 1 | 20.000 | 0.000 | *** |
| Asanang | AaSat006 | 1 | 0.013 | 0.909 | ns |
| Asanang | AaSat008 | Monomorphic | |  |  |
| Asanang | AaSat14 | 3 | 1.254 | 0.740 | ns |
| Asanang | AaSat020 | Monomorphic | |  |  |
| Asanang | AaSat040 | 1 | 12.735 | 0.000 | *** |
| Asanang | AaSat044 | 1 | 0.171 | 0.679 | ns |
| Asanang | AaSat053 | 6 | 0.900 | 0.989 | ns |
| Asanang | AaSat065 | 3 | 40.000 | 0.000 | *** |
| Asanang | AaGSat019 | Monomorphic | |  |  |
| Asanang | AaGSat026 | Monomorphic | |  |  |
| Asanang | AaGSat037 | 1 | 0.140 | 0.709 | ns |
| Selsela | AaSat001 | Monomorphic | |  |  |
| Selsela | AaSat002 | 3 | 9.074 | 0.028 | * |
| Selsela | AaSat006 | Monomorphic | |  |  |
| Selsela | AaSat008 | Monomorphic | |  |  |
| Selsela | AaSat14 | 1 | 0.444 | 0.505 | ns |
| Selsela | AaSat020 | Monomorphic | |  |  |
| Selsela | AaSat040 | 1 | 0.194 | 0.659 | ns |
| Selsela | AaSat044 | 1 | 2.591 | 0.107 | ns |
| Selsela | AaSat053 | 6 | 3.556 | 0.736 | ns |
| Selsela | AaSat065 | Monomorphic | |  |  |
| Selsela | AaGSat019 | 1 | 10.000 | 0.002 | ** |
| Selsela | AaGSat026 | Monomorphic | |  |  |
| Selsela | AaGSat037 | Monomorphic | |  |  |
| WWS1 | AaSat001 | 6 | 26.641 | 0.000 | *** |
| WWS1 | AaSat002 | 21 | 25.336 | 0.233 | ns |
| WWS1 | AaSat006 | 1 | 4.735 | 0.030 | * |
| WWS1 | AaSat008 | 6 | 10.734 | 0.097 | ns |
| WWS1 | AaSat14 | 1 | 15.000 | 0.000 | *** |
| WWS1 | AaSat020 | 21 | 51.917 | 0.000 | *** |
| WWS1 | AaSat040 | 21 | 48.503 | 0.001 | *** |
| WWS1 | AaSat044 | 6 | 37.043 | 0.000 | *** |
| WWS1 | AaSat053 | 6 | 19.096 | 0.004 | ** |
| WWS1 | AaSat065 | 6 | 20.595 | 0.002 | ** |
| WWS1 | AaGSat019 | 1 | 18.000 | 0.000 | *** |
| WWS1 | AaGSat026 | 3 | 24.000 | 0.000 | *** |
| WWS1 | AaGSat037 | 1 | 0.529 | 0.467 | ns |
| Agropetal | AaSat001 | 1 | 18.000 | 0.000 | *** |
| Agropetal | AaSat002 | 6 | 16.357 | 0.012 | * |
| Agropetal | AaSat006 | 1 | 0.014 | 0.906 | ns |
| Agropetal | AaSat008 | Monomorphic | |  |  |
| Agropetal | AaSat14 | 1 | 0.055 | 0.814 | ns |
| Agropetal | AaSat020 | Monomorphic | |  |  |
| Agropetal | AaSat040 | 1 | 16.191 | 0.000 | *** |
| Agropetal | AaSat044 | 1 | 4.281 | 0.039 | * |
| Agropetal | AaSat053 | 1 | 8.183 | 0.004 | ** |
| Agropetal | AaSat065 | 1 | 20.000 | 0.000 | *** |
| Agropetal | AaGSat019 | 1 | 20.000 | 0.000 | *** |
| Agropetal | AaGSat026 | Monomorphic | |  |  |
| Agropetal | AaGSat037 | 1 | 1.469 | 0.225 | ns |
| Bhagmara | AaSat001 | Monomorphic | |  |  |
| Bhagmara | AaSat002 | 3 | 14.026 | 0.003 | ** |
| Bhagmara | AaSat006 | Monomorphic | |  |  |
| Bhagmara | AaSat008 | Monomorphic | |  |  |
| Bhagmara | AaSat14 | Monomorphic | |  |  |
| Bhagmara | AaSat020 | Monomorphic | |  |  |
| Bhagmara | AaSat040 | 1 | 9.000 | 0.003 | ** |
| Bhagmara | AaSat044 | 1 | 6.043 | 0.014 | * |
| Bhagmara | AaSat053 | 1 | 15.000 | 0.000 | *** |
| Bhagmara | AaSat065 | 1 | 10.000 | 0.002 | ** |
| Bhagmara | AaGSat019 | Monomorphic | |  |  |
| Bhagmara | AaGSat026 | Monomorphic | |  |  |
| Bhagmara | AaGSat037 | Monomorphic | |  |  |
|  |  |  |  |  |  |
| **Key: ns=not significant, * P<0.05, ** P<0.01, *** P<0.001** | | | | |  |
